# Supplementary material for: Combining genotypes and T cell receptor distributions to infer genetic loci determining V(D)J recombination probabilities
Source: eLife. 2022 Mar 22;11:e73475. doi: 10.7554/eLife.73475 (PMC8940181; doi:10.7554/eLife.73475)
Supplement: Transparent reporting form [file elife-73475-transrepform1.pdf]

Westbrook Centre
Milton Road
Cambridge CB4 1YG, UK
P 01223 855340
W elifesciences.org
T @elife

eLife's transparent reporting form

We encourage authors to provide detailed information *within their submission* to facilitate the interpretation and replication of experiments. Authors can upload supporting documentation to indicate the use of appropriate reporting guidelines for health-related research (see EQUATOR Network), life science research (see the BioSharing Information Resource), or the ARRIVE guidelines for reporting work involving animal research. Where applicable, authors should refer to any relevant reporting standards documents in this form.

If you have any questions, please consult our Journal Policies and/or contact us: editorial@elifesciences.org.

Sample-size estimation

- You should state whether an appropriate sample size was computed when the study was being designed
- You should state the statistical method of sample size computation and any required assumptions
- If no explicit power analysis was used, you should describe how you decided what sample (replicate) size (number) to use

Please outline where this information can be found within the submission (e.g., sections or figure legends), or explain why this information doesn't apply to your submission:

The size of the discovery cohort (N=398) was determined by data availability (TCR repertoires and associated SNP genotyping data) from previous studies.

The size of the validation cohort (N=94) was determined by PBMC and DNA sample availability from ongoing studies. No explicit power analyses was conducted prior to the study, rather, all possible samples were utilized for maximum power possible.

Further details about cohort data availability and preparation can be found in the Methods section beginning on page 19.

Replicates

- You should report how often each experiment was performed
- You should include a definition of biological versus technical replication
- The data obtained should be provided and sufficient information should be provided to indicate the number of independent biological and/or technical replicates
- If you encountered any outliers, you should describe how these were handled
- Criteria for exclusion/inclusion of data should be clearly stated
- High-throughput sequence data should be uploaded before submission, with a private link for reviewers provided (these are available from both GEO and ArrayExpress)

eLife Sciences Publications, Ltd is a limited liability non-profit non-stock corporation incorporated in the State of Delaware, USA, with company number 5030732, and is registered in the UK with company number FC030576 and branch number BR015634 at the address Westbrook Centre, Milton Road, Cambridge CB4 1YG.

Please outline where this information can be found within the submission (e.g., sections or figure legends), or explain why this information doesn't apply to your submission:

The discovery cohort is based on pre-existing datasets. The validation cohort has 94 samples, and each library prep was generated/processed once. These samples represent biological replicates, defined here as measurements of biologically distinct samples. This information can be found in the "Validation cohort dataset" subsection within the Methods section on page 19.

Data processing for discovery and validation cohort TCR repertoire and SNP genotyping data and model setup are described in detail in the Methods section. Briefly, for all of our analyses, we excluded TCR repertoires with excessively small numbers of sequences. Further, we excluded SNP data with a minor allele frequency in the sample population of less than 0.05.

All data is publicly available and links or accession numbers are provided in the "Data availability" section, underneath "Additional Files".

Statistical reporting

- Statistical analysis methods should be described and justified
- Raw data should be presented in figures whenever informative to do so (typically when N per group is less than 10)
- For each experiment, you should identify the statistical tests used, exact values of N, definitions of center, methods of multiple test correction, and dispersion and precision measures (e.g., mean, median, SD, SEM, confidence intervals; and, for the major substantive results, a measure of effect size (e.g., Pearson's r, Cohen's d)
- Report exact p-values wherever possible alongside the summary statistics and 95% confidence intervals. These should be reported for all key questions and not only when the p-value is less than 0.05.

Please outline where this information can be found within the submission (e.g., sections or figure legends), or explain why this information doesn't apply to your submission:

We provide information about statistical analyses within the main text (e.g. bottom of page 14, line 281), in the figure legends (e.g. Figure 8), and in the Methods section. For each GWAS for each repertoire feature of interest, we have also included a supplementary file containing additional information for all SNPs which were classified as significant using the corresponding model and Bonferroni corrected significance cut-off described in Table 1.

(For large datasets, or papers with a very large number of statistical tests, you may upload a single table file with tests, Ns, etc., with reference to sections in the manuscript.)

Group allocation

- Indicate how samples were allocated into experimental groups (in the case of clinical studies, please specify allocation to treatment method); if randomization was used, please also state if restricted randomization was applied

- Indicate if masking was used during group allocation, data collection and/or data analysis

Please outline where this information can be found within the submission (e.g., sections or figure legends), or explain why this information doesn't apply to your submission:

Interventional clinical studies are not included in this research, thus allocation to experimental groups does not apply here.

Detailed methods regarding PCA cluster grouping used to correct for population-substructure-related biases due to ancestry in our GWAS analyses can be found within the "Ancestry-informative PCA cluster classification" section within the Methods section (page 27).

Additional data files ("source data")

- We encourage you to upload relevant additional data files, such as numerical data that are represented as a graph in a figure, or as a summary table
- Where provided, these should be in the most useful format, and they can be uploaded as "Source data" files linked to a main figure or table
- Include model definition files including the full list of parameters used
- Include code used for data analysis (e.g., R, MatLab)
- Avoid stating that data files are "available upon request"

Please indicate the figures or tables for which source data files have been provided:

We are providing a supplementary source data file (in .txt format) for all main figures.

eLife Sciences Publications, Ltd is a limited liability non-profit non-stock corporation incorporated in the State of Delaware, USA, with company number 5030732, and is registered in the UK with company number FC030576 and branch number BR015634 at the address Westbrook Centre, Milton Road, Cambridge CB4 1YG.
